# 固相萃取-超高效液相色谱-串联质谱法同时测定动物源性食品中11种禁限兽药及代谢物

**DOI:** 10.3724/SP.J.1123.2020.05012

**Published:** 2021-04-08

**Authors:** Bolin LIU, Jian XIE, Ziwei ZHAO, Xiuli WANG, Xiaomei SHAN

**Affiliations:** 安徽省疾病预防控制中心, 安徽 合肥, 230601; Anhui Provincial Center for Disease Control and Prevention, Hefei 230601, China; 安徽省疾病预防控制中心, 安徽 合肥, 230601; Anhui Provincial Center for Disease Control and Prevention, Hefei 230601, China; 安徽省疾病预防控制中心, 安徽 合肥, 230601; Anhui Provincial Center for Disease Control and Prevention, Hefei 230601, China; 安徽省疾病预防控制中心, 安徽 合肥, 230601; Anhui Provincial Center for Disease Control and Prevention, Hefei 230601, China; 安徽省疾病预防控制中心, 安徽 合肥, 230601; Anhui Provincial Center for Disease Control and Prevention, Hefei 230601, China

**Keywords:** 超高效液相色谱-串联质谱, 固相萃取, 基质效应, 同位素内标, 兽药残留, 代谢物, 动物源性食品, ultra performance liquid chromatography-tandem mass spectrometry(UPLC-MS/MS), solid phase extraction (SPE), matrix effect (ME), isotope internal standard, veterinary drug residues, metabolites, animal-derived foods

## Abstract

建立了固相萃取-超高效液相色谱-串联质谱(SPE-UPLC-MS/MS)同时检测鸡蛋、液态奶、鸡肉及淡水鱼中4类(氯霉素类、硝基咪唑类、林可酰胺类与大环内酯类)8种禁限兽药与3种代谢物残留的分析方法。对样品前处理及色谱条件进行优化,样品经0.1 mol/L pH 9.0的磷酸盐缓冲液水解分散,乙腈提取,提取液经乙酸乙酯萃取后浓缩至近干,残留物用0.3 mL甲醇溶解,再加入5.7 mL磷酸盐缓冲液,混匀,溶解液经Oasis HLB固相萃取柱净化后上机分析,以甲醇与0.1%甲酸水溶液作为流动相进行梯度洗脱,经ACQUITY UPLC BEH C18色谱柱(100 mm×2.1 mm, 1.7 μm)分离,采用ESI^+^和ESI^-^电离,在多反应监测模式下采集,同位素内标法定量。结果表明,11种待测物在色谱柱上完全基线分离,在各自范围内线性关系良好,相关系数(*R*^2^)>0.99,方法的检出限(LOD)为0.050~0.50 μg/kg,定量限(LOQ)为0.20~1.5 μg/kg。各待测物的平均回收率为65.3%~108%,相对标准偏差(RSD)为0.40%~21%。将该方法应用于市售样品中上述4类兽药残留的常规监测,其阳性样本的检测结果与标准方法测定结果无显著性差异。该方法灵敏度高,稳定性好,定量准确,适用于动物源性食品中多种禁限兽药及代谢物残留的同时快速测定。

## Abstract (en)

Chloramphenicols, nitroimidazoles, lincosamides, and macrolides are common antibiotics used in veterinary medicine. Overdoses of these drugs will lead to residual substances in animal-derived foods and accumulate in the body through the food chain, thereby exerting adverse effects on human health. Therefore, regulation of veterinary drug levels is imperative to ensure the quality of animal-derived foods and safeguard the health of consumers. In this study, a method based on ultra performance liquid chromatography-tandem mass spectrometry coupled with solid phase extraction (SPE-UPLC-MS/MS) was developed for the simultaneous determination of eight prohibited and restricted veterinary drugs and three metabolite residues across four categories (chloramphenicols, nitroimidazoles, lincosamides, and macrolides) in eggs, liquid milk, chicken, and freshwater fish. The main factors affecting the response, recovery, and sensitivity of the method, such as the type and pH values of the extraction solvent, dilution solution for the analytes, type of chromatographic column, and type and proportion of the mobile phase, were optimized during sample pretreatment and instrument analysis. The samples were hydrolyzed and dispersed in 0.1 mol/L phosphate buffer solutions (pH 9.0) and extracted with acetonitrile. The extract was further extracted using ethyl acetate. After centrifugation, the supernatant ethyl acetate was concentrated to near dryness in nitrogen below 40 ℃. The residue was dissolved in 0.3 mL methanol, followed by the addition of 5.7 mL phosphate buffer solution. After shaking, the solutions were purified and enriched on an Oasis HLB SPE column. The target analytes were separated on an ACQUITY UPLC BEH C18 chromatographic column (100 mm×2.1 mm, 1.7 μm) at a column temperature of 40 ℃ with a flow rate of 0.4 mL/min. The injection volume was 10 μL. Gradient elution was carried out with methanol and 0.1% formic acid aqueous solution as the mobile phases. Multiple reaction monitoring (MRM) was conducted in the positive and negative electrospray ionization modes. The isotope internal standard method was used for quantitative analysis. Under optimal conditions, each analyte showed a good linear relationship in each range, and the correlation coefficient (*R*^2^) was greater than 0.99. The limits of detection (LODs) ranged from 0.050 to 0.50 μg/kg, and the limits of quantification (LOQs) ranged from 0.20 to 1.5 μg/kg. With eggs, freshwater fish, chicken, and liquid milk as the matrix samples, the recoveries in spiked blank samples were determined at different addition levels in compliance with the current legislation. The average recoveries of the 11 analytes were 65.3% to 108%. The relative standard deviations (RSDs) were between 0.40% and 21%. The matrix effects of the analytes were between 0.0124% and 46.80% in four different samples after purification on the Oasis HLB column. The practicality of the proposed approach for routine analyses of the eight prohibited and restricted veterinary drugs, and three metabolite residuals was evaluated by applying it to the determination of these compounds in animal-derived food samples. The samples, including 80 eggs, 80 chicken, 40 liquid milk, and 32 freshwater fish, were procured from a supermarket and a farm product market. The results of the positive samples were consistent with those observed with the standard methods. The method described herein is easy to operate, sensitive, and accurate. It is suitable for the simultaneous and rapid determination of various prohibited and restricted veterinary drug residues and metabolites in animal-derived foods.

氯霉素类、硝基咪唑类、林可酰胺类与大环内酯类为常见的抗生素药物,广泛用于养殖过程中多种疾病的预防和细菌性感染类的治疗。超量或超种类使用这些药物后,会残留于动物性食品中,并通过食物链在人体内蓄积,危害人类健康^[1]^,我国农业部第250号公告规定食品动物中禁止使用氯霉素、洛硝达唑等药物,国家标准GB 31650-2019规定了食品动物中甲砜霉素、氟苯尼考、氟苯尼考胺、林可霉素、红霉素等药物的最高残留限量(MRLs)。但食品中检出林可霉素、氯霉素、甲硝唑、红霉素及代谢物等药物残留事件有报道^[1,2,3,4]^。兽药残留是目前食品安全所面临的重大问题之一。

针对食品中多种兽药残留的检测,目前报道的方法有液相色谱-四极杆飞行时间质谱法(LC-QTOF/MS)^[5,6]^和液相色谱-四极杆/静电场轨道阱高分辨质谱法(LC-HRMS)^[7,8,9]^等,这些方法能够提供待测物母离子和碎片离子的精确质量数,降低了近似质量数的干扰,更好地避免了假阳性的发生,是食品中多种兽药残留快速定性筛查的首选方法。不足的是,这些快速定性筛查方法的定量效果欠佳^[10]^,且仪器设备昂贵。超高效液相色谱-串联质谱法(UPLC-MS/MS)因分析效率高、灵敏度高等优势,成为抗生素残留分析的主流检测方法^[11]^。尽管采用UPLC-MS/MS测定禽蛋、水产品、蜂蜜、牛奶及奶粉等食品中多种兽药残留有较多文献报道^[4,12-19]^,但样品前处理方法各不相同,以QuEChERS法、OasisPRiME HLB固相萃取柱净化等方法居多,这两种前处理方法具有简单、快速的优势,能解决同一样品中多种兽药净化处理。但吸附剂在吸附极性与非极性杂质的同时也会吸附待测物,导致部分待测物回收率低,影响定量的准确性。而且现已报道的检测方法中,多是检测药物原型,涉及代谢物的检测方法不多。而文献^[3]^报道红霉素在酸性条件下不稳定,易降解成红霉素A烯醇醚和脱水红霉素A,这些降解产物被认为是使人体产生胃肠道反应的主要原因^[20]^,所以同时检测红霉素及其代谢物具有重要意义。另外,国家食品安全风险监测常规项目包含林可霉素、红霉素、甲硝唑、氯霉素类及代谢物等兽药残留,按现行标准方法及国家食品安全风险监测工作手册方法^[21]^,分为4次样品前处理及上机测定,耗费大量时间、人力、物力与财力。因此,迫切需要建立动物源性食品中这4类兽药残留及代谢物的同时检测方法。

针对上述存在的问题,本文以鸡蛋、液态奶、鸡肉及淡水鱼样品为基质,优化提取净化条件,考察基质效应的影响,结合同位素内标稀释法,有效降低了基质效应,建立了SPE-UPLC-MS/MS同时定量测定氯霉素类、硝基咪唑类、林可霉素、红霉素共4类8种禁限兽药与3种代谢物的残留,实现了鸡蛋、液态奶、鸡肉及淡水鱼中多类兽药残留及代谢物的同时测定,解决了常规监测采用4种前处理及仪器分析方法的难题,为食品安全风险监测及隐患排查提供高效、便捷、准确可靠的分析方法。

## 1 实验部分

### 1.1 仪器与试剂

ACQUITY^TM^ UPLC超高压液相色谱仪、ZEVO TQ MS质谱仪、Acquity^TM^ UPLC BEH C18色谱柱(100 mm×2.1 mm, 1.7 μm)(美国Waters公司); VORTEX Multi Reax高速旋涡混匀器(德国Heidolph公司); Milli-Q超纯水系统(美国Millipore公司); Legend Mach 1.6R高速冷冻离心机(美国Thermo Fisher公司)。

氯霉素、甲砜霉素,纯度均大于97%,购自美国Sigma-Aldrich公司;甲硝唑、地美硝唑、洛硝达唑、林可霉素、氟苯尼考、氟苯尼考胺、氯霉素-D_5_、氟苯尼考-D_3_、甲砜霉素-D_3_、林可霉素-D_3_、地美硝唑-D_3_、洛硝达唑-D_3_纯度均大于95%,购自德国Dr. Ehrenstorfer公司;脱氧红霉素A、红霉素A烯醇醚、红霉素A、红霉素A-D_6_、甲硝唑-D_4_、氟苯尼考胺-D_3_纯度均大于95%,购自加拿大TRC公司;甲醇、乙腈、甲酸、乙酸乙酯(色谱纯,德国Merck公司);乙酸铵(色谱纯,美国Sigma-Aldrich公司);无水磷酸氢二钠(分析纯,国药集团化学试剂北京有限公司); *N*-丙基乙二胺(PSA)、C18填料(均为50 μm,美国Agilent公司); Oasis HLB固相萃取柱(200 mg/6 mL,美国Waters公司)。

### 1.2 溶液的配制

分别称取11种标准品及同位素内标适量,用甲醇溶解,并定容至刻度,配制成100 μg/mL的标准储备液与内标储备液,避光保存于-20 ℃冰箱。

用适量10%甲醇水溶液稀释上述标准储备液,混匀,分别配制成100 ng/mL的混合标准中间液与混合同位素内标溶液,现用现配。

准确移取上述混合标准中间溶液10、50、100、200、500、1000与5000 μL,移至10 mL容量瓶中,加入500 μL混合同位素内标溶液,用10%甲醇水溶液定容至刻度,配制成0.1、0.5、1.0、2.0、5.0、10.0与50.0 μg/L的系列混合标准溶液,现用现配。

### 1.3 样品前处理

样品采集于本省不同地区的超市与农贸市场,鸡肉与鱼类样品取肌肉部位,经高速组织捣碎机均匀绞碎,用四分法缩分出适量,鸡蛋样品去壳混合均匀。所有样品均分成2份,一份用于检测,另一份用于复检,加封作标记,于-20 ℃保存。

称取2.00 g(精确至0.01 g)试样,置于50 mL聚丙烯具塞离心管中,加入50 μL混合同位素内标溶液(100 ng/mL)后,静置30 min,先加入5 mL pH 9.0磷酸盐缓冲溶液,涡旋振荡5 min后,再加入5 mL乙腈,混匀后,以10000 r/min冷冻离心10 min,取上清液于另一个离心管中,加入10 mL乙酸乙酯,涡旋30 s,静置分层后,以10000 r/min冷冻离心10 min,取上层液体于15 mL聚丙烯离心管中,于40 ℃以下水浴中氮气吹至近干,残留物用0.3 mL甲醇溶解后,加入5.7 mL磷酸盐缓冲液,混匀,待净化。

Oasis HLB固相萃取预先依次用5 mL甲醇、5 mL水、5 mL pH 9.0的磷酸盐缓冲溶液淋洗活化,将上述待净化液转移至活化后的Oasis HLB固相萃取柱上,弃去流出液,加入5 mL纯水淋洗后抽干,随后用6 mL甲醇分2次洗脱,控制流速1~2 mL/min。洗脱液于40 ℃以下水浴氮气吹干,用1.0 mL 10%甲醇水(先加入100 μL甲醇溶解残渣,再加入900 μL超纯水)溶液定容,混匀,以10000 r/min冷冻离心5 min,取上清液,上机测定。

### 1.4 分析条件

1.4.1 色谱条件

色谱柱:Waters ACQUITY UPLC BEH C18柱(100 mm×2.1 mm, 1.7 μm);柱温:40 ℃;样品室温度:10 ℃;流速:0.4 mL/min;流动相A: 0.1%甲酸水溶液;流动相B:甲醇。梯度洗脱程序:0~3.0 min, 90%A~60%A; 3.0~3.5 min, 60%A~15%A; 3.5~4.0 min, 15%A~10%A; 4.0~4.5 min, 10%A~90%A; 4.5~6.0 min, 90%A。进样量:10 μL。

1.4.2 质谱条件

离子源:电喷雾电离(ESI)源;毛细管电压:2.50 kV(负离子)和4.0 kV(正离子);离子源温度:150 ℃;脱溶剂气温度:500 ℃;脱溶剂气流量:1000 L/h;碰撞气流量:0.13 mL/min;多反应监测(MRM)模式。待测物的母离子、子离子及对应的碰撞能量、锥孔电压等质谱参数见表1。

**表 1:** 11种待测物及9种同位素内标的质谱参数

Analyte	ESI	*t*_R_/ min	Parent ion (*m/z*)	Product ions (*m/z*)	Collision energies/eV	Cone voltage/ V	IS
Florfenicol amine (氟苯尼考胺)	+	0.84	248.1	230.1^*^/130.1	12/24	30	florfenicol amine-D_3_
Metronidazole (甲硝唑)	+	1.49	172.1	128.1^*^/82.0	14/22	30	metronidazole-D_4_
Ronidazole (洛硝达唑)	+	1.55	201	140.0^*^/55.1	12/20	24	ronidazole-D_3_
Dimetridazole (地美硝唑)	+	1.68	142.1	96.0^*^/81.0	16/22	36	dimetridazole-D_3_
Thiamphenicol (甲砜霉素)	-	2.2	354	185.0^*^/290.1	20/12	30	thiamphenicol-D_3_
Lincomycin (林可霉素)	+	2.43	407.4	126.1^*^/359.3	28/20	52	lincomycin-D_3_
Florfenicol (氟苯尼考)	-	2.89	356	185.0^*^/336.1	18/10	36	florfenicol-D_3_
Chloramphenicol (氯霉素)	-	3.71	321	257.0/152.0^*^	10/20	30	chloramphenicol-D_5_
Erythromycin A (红霉素A)	+	4.25	734.5	158.1^*^/576.5	28/20	48	erythromycin A-D_6_
Erythromycin A enol ether	+	4.28	716.4	158.1^*^/83.0/558.3	30/42/20	44	erythromycin A-D_6_
(红霉素A烯醇醚)							
Anhydro erythromycin A (脱氧红霉素A)	+	4.42	716.4	158.1^*^/558.3	30/16	38	erythromycin A-D_6_
Florfenicol amine-D_3_ (氟苯尼考胺-D_3_)	+	0.83	251.2	233.1^*^/130.2	12/24	30	
Metronidazole-D_4_ (甲硝唑-D_4_)	+	1.49	176.2	128.1^*^/82.0	16/26	30	
Ronidazole-D_3_ (洛硝达唑-D_3_)	+	1.55	204	143.0^*^/58.0	20/12	24	
Dimetridazole-D_3_ (地美硝唑-D_3_)	+	1.68	145	99.0^*^/83.0	22/16	36	
Thiamphenicol-D_3_ (甲砜霉素-D_3_)	-	2.2	357.1	188.1^*^/293.1	20/12	40	
Lincomycin-D_3_ (林可霉素-D_3_)	+	2.43	410.4	129.1^*^/362.3	28/20	52	
Florfenicol-D_3_ (氟苯尼考-D_3_)	-	2.89	359.1	188.1^*^/339.1	18/10	36	
Chloramphenicol-D_5_(氯霉素-D_5_)	-	3.71	326	157.0^*^/262.0	18/12	30	
Erythromycin A-D_6_ (红霉素A-D_6_)	+	4.25	740.5	164.1^*^/582.5	32/20	48	

* Quantitative ion.

## 2 结果与讨论

### 2.1 定容溶剂的选择

用10%~50%(v/v)乙腈水溶液、10%~50%(v/v)甲醇水溶液稀释标准溶液,考察溶剂对待测物响应值与色谱峰形的影响。结果表明,随着甲醇或乙腈体积分数的增加,氟苯尼考胺响应和峰形逐渐变差,出现峰伸舌与拖尾现象,当体积分数超过40%时,所有待测物均有响应不稳定且峰形展宽的现象。考虑当甲醇或乙腈体积分数为10%时,各待测物的响应高,色谱峰形尖锐、对称。以10%甲醇为溶剂时,红霉素A烯醇醚与脱氧红霉素A达到基线分离,且11种待测物的响应值及峰形也较用10%乙腈水溶液时好。因此最终选择10%(v/v)甲醇水溶液作为标准及样品的定容溶剂。

### 2.2 色谱条件的优化

实验对流动相进行了优化。分别选择甲醇与乙腈为有机相,同时在水相中分别加入甲酸、氨水和乙酸铵,考察待测物的分离效果、色谱峰形、峰高及响应情况。结果发现,以纯水或纯水中加入乙酸铵为水相时,有机相无论是乙腈还是甲醇,林可霉素的峰形均较差,响应较低,需要在水相中加入酸、碱调节pH值来改善峰形。以乙腈作为有机相、水相中加入氨水时,红霉素A、红霉素A烯醇醚与脱氧红霉素A的峰形较差,有伸舌与拖尾现象,且响应低,同时发现,氨水-乙腈作为流动相虽能够增加负离子模式扫描模式下氯霉素、氟甲砜霉素、氟苯尼考等物质的信号强度,但是也容易使氟苯尼考的内标产生同位素峰效应,使内标的平均回收率增强4.5倍,当采用内标法定量时,往往会造成高浓度的实际样品结果偏低。而以甲醇作为有机相,并在水相中加入氨水时,除脱水红霉素峰形较用乙腈时有所改善外,红霉素A与红霉素A烯醇醚的峰形依然存在伸舌与拖尾现象,且林可霉素、氯霉素、氟甲砜霉素、氟苯尼考与甲硝唑等药物的峰形较用乙腈时差,影响准确定量。虽有文献^[4]^报道,碱性流动相条件下甲硝唑和林可霉素的响应值分别是酸性流动相条件下的7.5和5.4倍,但考虑同时分析红霉素A及其代谢物,水相中加入氨水不适合。因此,选择水相中加入甲酸,对于在负离子模式下扫描的氯霉素、氟甲砜霉素、氟苯尼考等物质来说,甲酸提供的H^+^抑制了这些化合物的解离,影响离子化效率,但能够促进[M+H]^+^峰形成的同时抑制目标化合物[M+Na]^+^峰的形成^[18]^,对于正离子模式来说,能够提高离子化效率,提高灵敏度,故选择0.1%甲酸水溶液作为水相。以甲醇为有机相时,各待测物的峰形均较乙腈时好,响应也更高,且红霉素A烯醇醚与脱氧红霉素A完全能够达到基线分离。因此最终确定0.1%甲酸水溶液与甲醇作为流动相。优化后的11种禁限兽药物及代谢物的MRM色谱图见图1。

**图 1:**
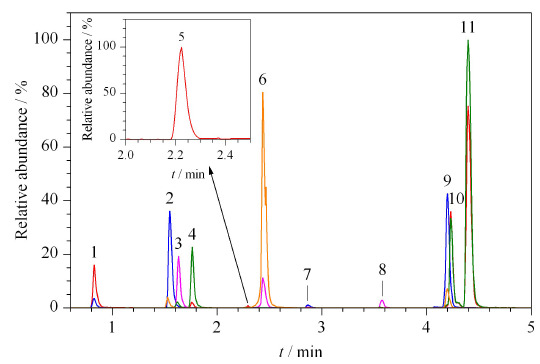
11种禁限兽药及代谢物的总离子流色谱图

### 2.3 前处理条件的优化

2.3.1 提取液及pH值的优化以阴性的鸡蛋样品为实验对象,加入11种禁限兽药及代谢物标准溶液,使其在样品中含量为10.0 ng,经4%氯化钠水溶液分散、加入乙腈沉淀蛋白质后,用乙酸乙酯提取,实验发现,红霉素A及代谢物和林可霉素的提取效率低,加标回收率小于4%,待测物的化学性质不同,不同的提取液影响了提取效果。据文献^[3]^报道,红霉素A在酸性条件下不稳定,在中性及弱碱性条件下稳定,故选择pH 7.0的磷酸盐缓冲液作为最佳提取液提取蜂蜜中红霉素A及代谢物。张晓艺等^[4]^发现在pH 10.0的碳酸盐缓冲液作为提取液的条件下,采用乙酸乙酯同时萃取蜂蜜中的氯霉素、甲硝唑和林可霉素,萃取效果较好。陈兴连等^[17]^研究认为碱性溶液作为提取液时,不仅能很好的分散样品,增加与有机溶剂接触面积,充分提取药物,同时碱溶液还能促进药物与结合蛋白质解离,有利于提高提取效率。综合上述学者的报道,本文选择pH 6.0~11.0的磷酸盐缓冲液作为萃取条件,考察不同pH值下,乙酸乙酯对11种待测物提取的效率,结果见图2。

**图 2:**
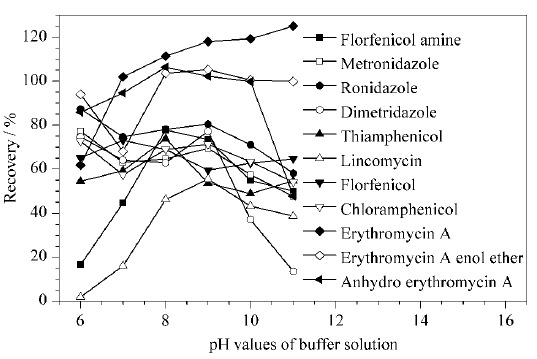
磷酸盐缓冲液的pH值对11种禁限兽药及代谢物提取效率的影响

结果表明,提取液的pH值对林可霉素提取效率的影响较明显,随着提取液pH值的增加,林可霉素的回收率逐渐增加,pH为9.0时回收率最高,当pH大于9.0时,林可霉素与地美硝唑的回收率降低。氯霉素类及代谢物、甲硝唑与洛硝达唑的提取效率受pH值影响不大,平均回收率为64.4%,红霉素及代谢物在pH为8.0~11.0的响应值最高,且比较稳定。综合考虑,本实验选择0.1 mol/L pH 9.0的磷酸氢二钠溶液作为提取液。2.3.2 净化方法的选择多兽药残留的同时测定,样品前处理多采用QuEChERS法或吸附-洗脱模式的固相萃取法。QuEChERS法是通过吸附剂与样品中的杂质相互作用,以实现分析物的提取和净化,Oasis PRiME HLB固相萃取柱是一款无须活化的固相萃取柱,通过柱中的填料吸附剂样品中的杂质,以实现分析物的提取和净化,Oasis HLB柱、MCS柱与BRP柱为活化-洗脱型的固相萃取柱,MCS为混合型阳离子交换柱,BRP是含单分散的亲水疏水表面平衡反相吸附剂的萃取柱,填料是经过表面修饰改性的聚合物,引入了极性官能基团,可以用于分离极性和非极性物质。而不同净化方法的净化效果取决于待测物的性质与样品基质复杂程度。

**图 3:**
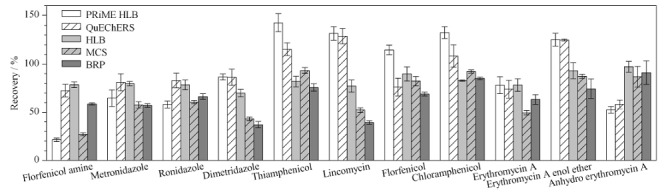
不同净化方式对11种禁限兽药及代谢物回收率的影响(*n*=3)

因此,本实验对比分析了不同净化方法的净化效果。结果如图3所示,采用Oasis PRiME HLB柱净化,氟苯尼考胺的回收率为21.4%,甲砜霉素、林可霉素及氯霉素的回收率均大于120%;采用QuEChERS法处理样品时,待测物的平均回收率为58.1%~129%,林可霉素、红霉素A烯醇醚的回收率大于120%,而脱氧红霉素A的回收率为58.1%;采用MCS柱净化,3种氯霉素类的平均回收率为82.3%~93.3%,但甲硝唑、林可霉素、地美硝唑与氟苯尼考胺的回收率比较低,分别为57.5%、52.3%、43.1%与27.1%,用BRP柱净化时,林可霉素与地美硝唑的回收率较低,分别为39.7%与37.0%。而采用Oasis HLB柱净化时,各待测物的平均回收率为69.9%~97.2%。综合考虑,本实验选择Oasis HLB柱为最佳净化固相萃取柱。

### 2.4 基质效应考察

基质效应(ME)是指基质中的共提取干扰物影响目标化合物的离子化^[6]^,从而影响目标物响应的现象。基质效应越强,方法的准确性越低^[18]^。本实验选择鸡蛋、鸡肉、液态奶与淡水鱼阴性样品为基质,采用1.3节描述的方法处理样品,获得空白基质提取液,分别制备基质匹配标准曲线与溶液标准曲线。根据文献^[22,23]^报道,采用ME=[基质匹配标准曲线的斜率/溶液标准曲线的斜率-1]×100%来计算11种禁限兽药及代谢物的基质效应,以评价不同基质中共提取干扰物对目标物响应的影响。如图4所示,红霉素烯醇酯在4种样品中均存在中等基质效应,|ME|为20%~50%,脱氧红霉素A在淡水鱼、鸡蛋与液态奶样品中存在中等基质效应,甲砜霉素在液态奶样品中为中等基质效应,其他待测物属于弱基质效应,|ME|为0~20%。因此,本实验采用基质匹配的同位素内标法加以校正。

**图 4:**
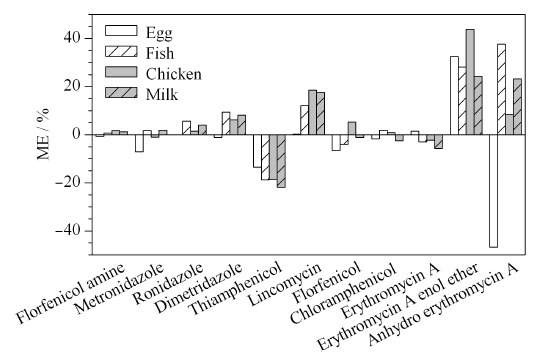
不同基质样品中11种禁限兽药及代谢物的基质效应

### 2.5 方法学验证

2.5.1 标准曲线与线性范围

以阴性淡水鱼为基质样品,按照1.3节描述提取和净化,氮气吹干后复溶获得基质溶液,用该基质溶液配制成0.1、0.5、1.0、2.0、5.0、10.0、20.0和50.0 μg/L的系列混合标准溶液,加入同位素内标溶液,使其质量浓度为5.0 μg/L,上机分析,内标法定量。以待测物与相对应同位素内标的峰面积之比为纵坐标(*y*),相应的质量浓度为横坐标(*x*, μg/L),绘制基质匹配标准曲线。结果表明,11种待测物在各自范围内线性关系良好,相关系数(*R*^2^)>0.99(见表2)。

**表 2:** 11种禁限兽药及代谢物的线性方程、线性范围、相关系数、检出限和定量限

Analyte	Linear equation	Linear range/(μg/L)	*R* ^2^	LOD/(μg/kg)	LOQ/(μg/kg)
Florfenicol amine	*y*=0.0898*x*-0.0035	0.1-50	0.9992	0.050	0.20
Metronidazole	*y*=0.1920*x*-0.0171	0.1-50	0.9972	0.050	0.20
Ronidazole	*y*=0.1535*x*-0.0172	0.1-50	0.9982	0.050	0.20
Dimetridazole	*y*=0.1894*x*-0.0155	0.1-50	0.9968	0.050	0.20
Thiamphenicol	*y*=0.0949*x*-0.0163	1.0-50	0.9927	0.50	1.5
Lincomycin	*y*=0.1311*x*-0.0088	0.1-50	0.9988	0.050	0.20
Florfenicol	*y*=0.0756*x*+0.0049	0.5-50	0.9971	0.25	0.80
Chloramphenicol	*y*=0.2078*x*+0.0066	0.5-50	0.9981	0.25	0.80
Erythromycin A	*y*=0.1283*x*-0.0116	0.2-50	0.9991	0.10	0.30
Erythromycin A enol ether	*y*=0.0705*x*-0.0099	0.2-50	0.9969	0.10	0.30
Anhydro erythromycin A	*y*=0.1414*x*-0.0336	0.2-50	0.9914	0.10	0.30

*y*: peak area ratio of the analytes to the internal standard; *x*: mass concentration, μg/L.

2.5.2 检出限及定量限

根据国家标准GB/T 27417-2017《化学分析方法确认和验证指南》的相关要求,分别添加不同低浓度的11种混合标准溶液到阴性样品中,对11种待测物进行测定,分别以3倍和10倍信噪比(*S/N*)对应的加标浓度定义检出限(LOD)与定量限(LOQ)。结果表明,该方法的LOD为0.050~0.50 μg/kg, LOQ为0.20~1.5 μg/kg(见表2)。

2.5.3 加标回收率及精密度

根据GB/T 27404-2008要求,对于食品中的禁用物质,分别添加约1、2、和10倍方法定量限水平的标准溶液于实际样品中,进行加标回收试验;已制定MRL的,应该在方法定量限、MRL选一合适点,未制定MRL的,在方法定量限、常见限量指标选一合适点进行加标回收试验。国家限量标准GB 31650-2019中规定,鸡肉、鸡蛋、牛奶与鱼肉中红霉素A的MRL值分别为100、50、40与200 μg/kg;鸡肉、鸡蛋、牛奶与鱼肉中林可霉素的MRL分别为200、50、150与100 μg/kg;鸡肉、牛奶与鱼肉中甲砜霉素的MRL均为50 μg/kg;鸡肉、鱼肉中氟苯尼考与氟苯尼考胺之和的MRL分别为100 μg/kg 和1000 μg/kg;我国农业部第250号公告规定食品动物中禁止使用氯霉素与洛硝达唑。根据上述加标规则,本实验选用空白本底的鸡蛋、鱼肉、鸡肉和液态奶为样品,添加11种待测物标准溶液,加标水平如表3所示,加入同位素内标混合液,上机测定,每个添加水平重复6次。结果表明,11种待测物的平均加标回收率为65.3%~108%,相对标准偏差为0.40%~21%。

**表 3:** 11种禁限兽药及代谢物在实际样品中的加标回收率和相对标准偏差(*n*=6)

Analyte	Spiked level/ (μg/kg)	Recoveries/%					RSDs/%				
		Egg	Fish	Chicken	Milk		Egg		Fish	Chicken	Milk
Florfenicol amine	0.2	97.9	89.7	98.4	94.0		11		13	10	14
	2.0	93.4	92.9	94.5	92.5		1.4		2.2	6.2	7.2
	100	97.1	100	94.0	92.3		6.8		2.4	11	4.9
Metronidazole	0.2	92.5	84.1	84.1	86.3		2.0		3.7	2.7	3.1
	2.0	91.0	83.8	77.9	81.5		3.3		1.5	1.1	3.3
	20	98.2	95.9	107	103		5.3		6.3	2.2	3.3
Ronidazole	0.2	92.5	95.4	98.7	93.7		4.5		5.0	2.8	6.8
	2.0	96.2	95.3	95.8	93.6		2.0		2.9	2.0	4.8
	20	94.4	93.5	107	94.8		1.7		1.9	4.0	7.3
Dimetridazole	0.2	99.0	105	100	102		7.8		2.8	2.1	5.1
	2.0	100	98.2	94.0	97.3		2.1		4.8	1.4	3.6
	20	96.5	97.7	83.8	104		6.7		7.2	5.2	6.9
Thiamphenicol	2.0	95.0	99.8	97.7	98.4		6.2		2.8	1.6	4.6
	10	102	97.4	101	103		0.40		1.2	2.7	2.4
	50	98.0	98.8	98.4	98.6		7.0		8.9	5.8	2.8
Lincomycin	0.2	102	91.9	89.7	85.8		6.2		9.9	4.1	3.3
	2.0	97.5	91.0	94.3	90.5		5.0		3.9	5.6	0.94
	50	83.6	76.7	77.8	95.0		1.5		5.4	10	8.6
Florfenicol	1.0	98.6	100	101	100		6.9		9.6	11	10
	2.0	102	99.1	99.5	98.3		2.5		2.07	2.90	3.3
	100	104	103	101	100		2.4		1.2	5.3	4.4
Chloramphenicol	1.0	102	107	108	106		3.4		2.0	1.7	0.76
	2.0	101	99.5	103	102		0.55		3.2	3.1	4.2
	10	103	101	103	107		5.3		3.0	4.2	2.0
Erythromycin A	0.3	87.5	93.5	90.7	94.7		4.7		4.2	1.1	5.5
	2.0	86.4	93.9	92.1	93.7		0.88		2.1	4.2	5.2
	40	104	102	100	99.7		5.5		0.8	4.2	5.6
Erythromycin A enol ether	0.3	65.3	104	84.4	82.1		11		4.2	15	8.1
	2.0	76.1	97.2	71.2	90.2		13		4.2	21	11
	10	86.9	99.9	83.2	90.2		14		3.9	10	7.0
Anhydro erythromycin A	0.3	101	86.3	98.7	94.4		2.7		5.5	9.3	6.0
	2.0	105	94.6	96.6	98.8		0.44		12	6.2	7.4
	10	105	95.0	102	108		8.8		16	4.4	0.85

### 2.6 实际样品的测定

采用该方法对市场上随机购买的80份鸡蛋、80份鸡肉、40份液态奶与32份淡水鱼进行检测,同时分别采用国标方法GB 29684-2013测定红霉素,GB/T 20762-2006测定林可霉素,GB/T 20744-2006测定硝基咪唑类,GB/T 20756-2006测定氯霉素类与SN/T 1865-2016测定氟苯尼考胺,考察本方法的可靠性。

结果表明,其中1份鸡蛋样品同时检出甲硝唑、氟苯尼考及其代谢物氟苯尼考胺,检出值分别为12.1/12.5 μg/kg(前者为本方法测定值/后者为标准方法测定值,下同)、7.52/7.06 μg/kg与12.3/12.4 μg/kg; 1份鸡蛋中检出氟苯尼考,检出值为3.23/3.03 μg/kg; 1份鸡蛋与1份鸡肉中分别检出甲硝唑,检出值为0.125/0.100 μg/kg与0.144/0.129 μg/kg。将该分析方法与标准方法所获取的阳性样本数据进行统计学分析,结果显示,两套分析方法无显著性差异(*t*=0.283, *P*=0.788)。可见本方法准确可靠。

## 3 结论

本文建立了固相萃取-超高效液相色谱-串联质谱同时测定动物源性食品中11种禁限兽药及代谢物的方法,结合同位素稀释技术,有效降低了实际样品的基质效应。该方法稳定性好,准确度高,适用于食品中多种痕量兽药残留及代谢物的同时检测。与现行国家标准方法相比,本方法能够实现不同类别的兽药残留及代谢物同时前处理与分析,缩短了检测周期,节约了检测成本,为例行监测提供支持。
